# Aestivation and hypoxia-related events share common silent neuron trafficking processes

**DOI:** 10.1186/1471-2202-13-39

**Published:** 2012-04-20

**Authors:** Giuseppina Giusi, Merylin Zizza, Rosa Maria Facciolo, Shit Fun Chew, Yuen Kwong Ip, Marcello Canonaco

**Affiliations:** 1 Comparative Neuroanatomy Laboratory, University of Calabria, 87030, Arcavacata di Rende (CS), Italy; 2 Natural Sciences, National Institute of Education, Nanyang Technological University, Singapore, Republic of Singapore; 3 Department of Biological Sciences, National University of Singapore, Singapore, Republic of Singapore

**Keywords:** Hypoxia, Glutamate, Neuroprotection, Apoptosis, qPCR, Chaperones

## Abstract

**Background:**

The availability of oxygen is a limiting factor for neuronal survival since low levels account not only for the impairment of physiological activities such as sleep-wake cycle, but above all for ischemic-like neurodegenerative disorders. In an attempt to improve our knowledge concerning the type of molecular mechanisms operating during stressful states like those of hypoxic conditions, attention was focused on eventual transcriptional alterations of some key AMPAergic silent neuronal receptor subtypes (GluR1 and GluR2) along with HSPs and HIF-1α during either a normoxic or a hypoxic aestivation of a typical aquatic aestivator, i.e. the lungfish (*Protopterus annectens*).

**Results:**

The identification of partial nucleotide fragments codifying for both AMPA receptor subtypes in *Protopterus annectens* displayed a putative high degree of similarity to that of not only fish but also to those of amphibians, birds and mammals. qPCR and *in situ* hybridization supplied a very high (*p* < 0.001) reduction of GluR1 mRNA expression in diencephalic areas after 6 months of aerial normoxic aestivation (6mAE). Concomitantly, high (*p* < 0.01) levels of HSP70 mRNAs in hypothalamic, mesencephalic and cerebellar areas of both 6mAE and after 6 months of mud hypoxic aestivation (6mMUD) were detected together with evident apoptotic signals. Surprisingly, very high levels of GluR2 mRNAs were instead detected in thalamic along with mesencephalic areas after 6 days of normoxic (6dAE) and hypoxic (6dMUD) aestivation. Moreover, even short- and long-term hypoxic states featured high levels of HIF-1α and HSP27 transcripts in the different brain regions of the lungfish.

**Conclusions:**

The distinct transcriptional variations of silent neurons expressing GluR1/2 and HSPs tend to corroborate these factors as determining elements for the physiological success of normoxic and hypoxic aestivation. A distinct switching among these AMPA receptor subtypes during aestivation highlights new potential adaptive strategies operating in key brain regions of the lungfish in relation to oxygen availability. This functional relationship might have therapeutic bearings for hypoxia-related dysfunctions, above all in view of recently identified silent neuron-dependent motor activity ameliorations in mammals.

## Background

Among the different environmental factors, oxygen proves to be a very critical element for the maintenance of cerebral homeostatic conditions [1]. It has been established that some vertebrates may tolerate prolonged hypoxia and high temperatures without damaging vital organs such as the brain [2]. In this category, aestivating turtles and fish are considered valuable models for clinical studies [3,4]. Aestivation is characterized by a consistent metabolic reduction accompanied by a decrease of temperature and a down-regulation of gas exchange and heart rate [5]. By surviving in cocoons for long periods, the aestivating lungfish *Protopterus annectens* unlike hibernators is protected against ammonia toxicity during drought periods above all for mud aestivators, which feature low oxygen levels [6]. Works have shown that the brain is a major target of oxygen depletion, as shown by brief hypoxia conditions reducing ATP levels and consequently membrane depolarization leading to neuronal cell death [7].

Under these conditions, the opening of α-amino-3-hydroxy-5-methyl-4-isoxazole-propionic acid (AMPA) receptor channels not only mediates fast excitatory neurotransmission in many synapses [8], but also accelerates hypoxia-dependent death [9]. In this case, Ca^2+^ flowing through AMPA receptor channels tends to exert a critical role on apoptosis during epilepsy, hypoxia-ischemia and Alzheimer disease [10,11]. Among the four AMPAergic subtypes (GluR1-4), those recognized as silent neurons (GluR1 and GluR2) are able to modulate synaptic strength by being inserted at post-synaptic containing NMDA receptor sites and so these subtypes may regulate dendritic morphology and synaptic transmission via phosphorylative processes during development as well as in pathological conditions [12]. Interestingly under hypoxia conditions, AMPA synaptic activities require the functional involvement of some neuroprotective factors, such as heat shock proteins (HSPs) and hypoxia-inducible factor-1 alpha (HIF-1α) [13,14]. HIF-1α is an important transcriptional factor coordinating adaptive responses against hypoxia in mammals [15] and in fish [16] and so by improving animal’s ability to resist to low oxygen conditions, this factor may avoid cellular damages [17] through the recruitment of other neuroprotective factors such as p53 and HSPs [18]. HSPs are highly conserved cell proteins responding to stressful conditions, such as heat shock, hypoxia and metabolic abnormalities [19,20]. At the brain level, HSP-dependent changes following oxygen deprivation are tightly correlated to ion channel plasticity of olfactory cortical cells, where these chaperones preserve glutamatergic synaptic transmission [21]. From a functional point of view, HIF-1α promotes the activation of small HSPs such as HSP27 during early ischemic insults [22] while other HSPs, and precisely HSP70, are strongly associated with long-term plasticity events at the synaptic level [23,24].

On this basis, it was our intention to identify a temporal relationship of the expression capacity of the silent neurons GluR1 and/or GluR2 to that of the activation of HIF-1α along with HSP27 and HSP70 under normoxic and hypoxic aestivating conditions of the lungfish. The selection of this fish as our experimental model is based on its adaptive capacity to oxygen deprivation by entering into a quiescent aestivating state. The identification of GluR1 and GluR2 mRNA expression patterns overlapping with the above neuroprotective factors might have potential therapeutic application during hypoxia–dependent neurodegenerative disorders, such as ischemia.

## Results

### GluR1 and GluR2: Molecular identification and distribution pattern

**Figure 1:**
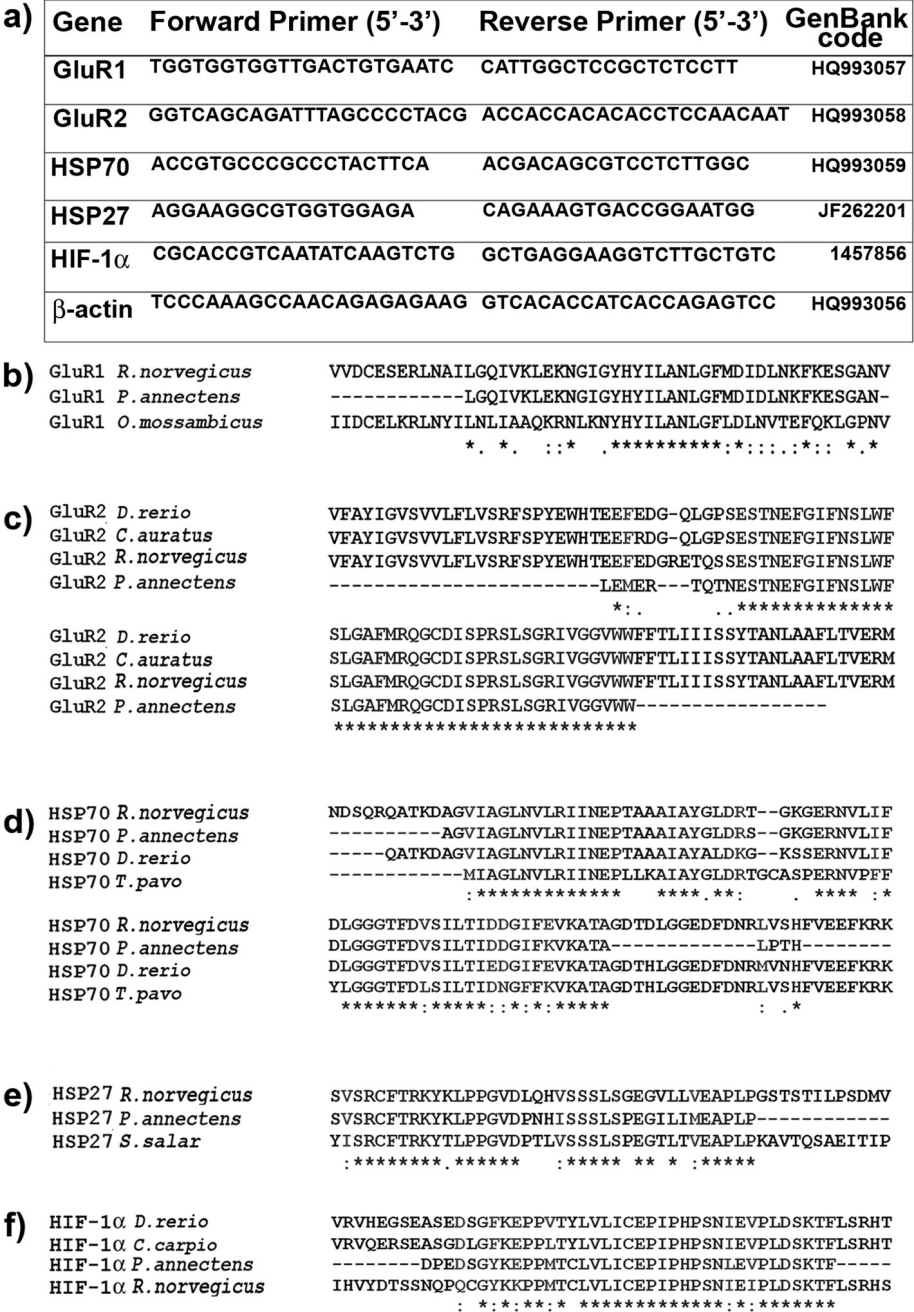
**Molecular identification of GluR1, GluR2, HSP70, HSP27 and HIF-1α in*Protopterus annectens.*** Primers sequences used for the amplification of AMPA receptor subtypes and protective factors were reported in the table **(a)**. The partial sequences obtained in *Protopterus annectens* have been aligned with GluR1 protein sequences **(b)** of *Rattus norvegicus* [GenBank: CAA35050] and *Oreochromis mossambicus* [GenBank: AAL34309], while in the case of GluR2 **(c)** with those of *Rattus norvegicus* [GenBank: AAD51284], *Carassius auratus* [GenBank: CAP08035] and *Danio rerio* [GenBank: AAQ08956]. Similarly, HSP70 partial protein sequence of *Protopterus annectens***(d)** was aligned to heterologs of *Rattus norvegicus* [GenBank: AAA17441], *Danio rerio* [GenBank: AAC17598] and *Thalassoma pavo* [GenBank: ABN58790], HSP27 **(e)** with those of *Rattus norvegicus* [GenBank: AAA41353] and *Salmo salar* [GenBank: ACI68354] and HIF-1α **(f)** with sequences of *Rattus norvegicus* [GenBank: AAD24413], *Danio rerio* [GenBank: AAI65712] and *Cyprinus carpio* [GenBank: ABV59209].

**Figure 2:**
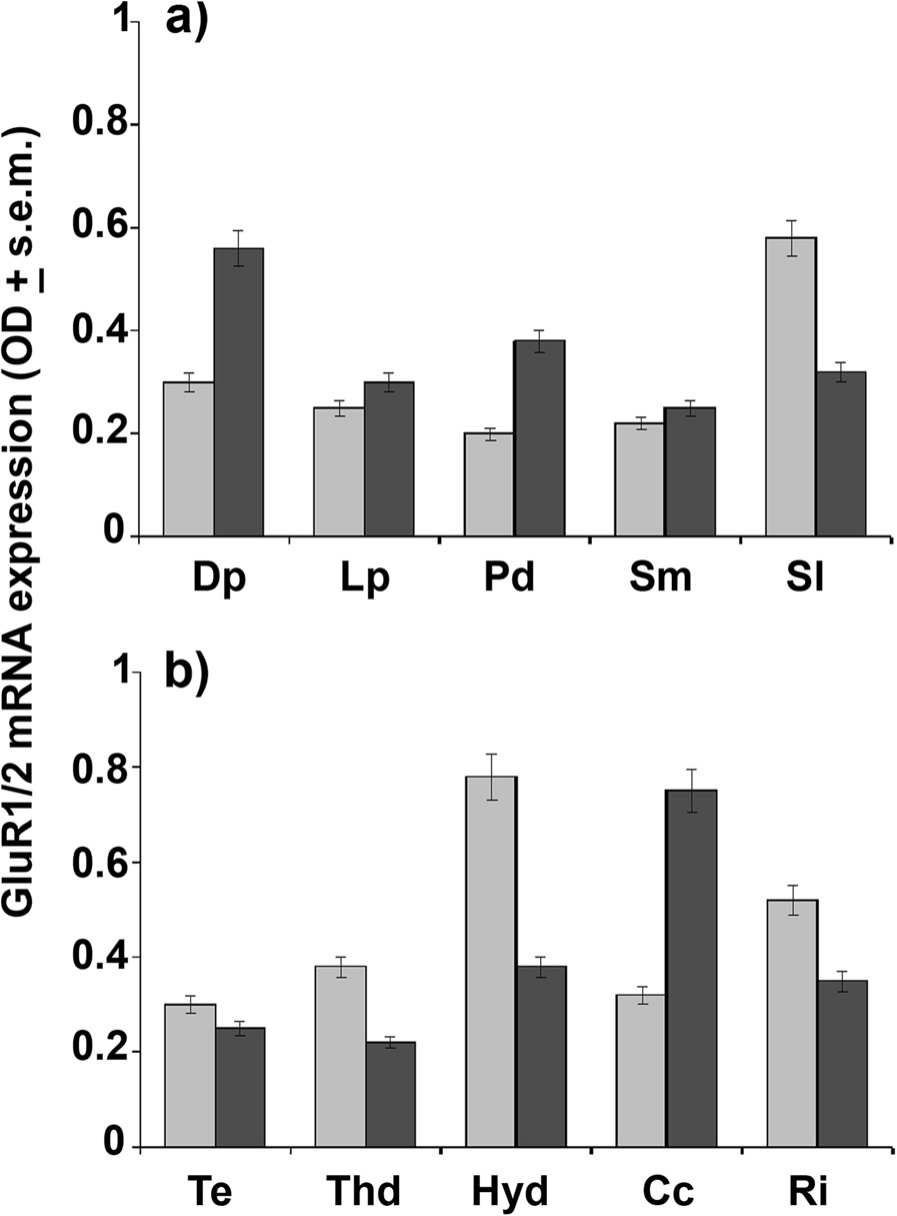
**GluR1 and GluR2 mRNA expression in FW conditions obtained by*in situ*hybridization method.** The mRNA levels (OD ± s.e.m.) of GluR1 (light grey) and GluR2 (dark grey) were evaluated in anterior **(a)** and posterior **(b)** areas of lungfish maintained under FW conditions. For abbreviations check list.

In this work, the application of specific primers designed on rat heterologs (Figure 1a) permitted us to identify, for the first time, a partial coding sequence of putative GluR1 [GenBank: HQ993057] and GluR2 [GenBank: HQ993058] of AMPA receptor complex along with β-actin [GenBank: HQ993056] in *Protopterus annectens*. These sequences showed a high nucleotide identity (>80%) with not only the corresponding sequences of AMPA receptor subtypes of *Rattus norvegicus* [GenBank: X17184; AF164344] but also with those of fish such as GluR1 of *Oreochromis mossambicus* [GenBank: L49498] (Figure 1b) and GluR2 of *Carassius auratus* (AM886311) and of *Danio rerio* [GenBank: AF525743] (Figure 1c). In a first case, a distinct anterior-posterior expression gradient of GluR1 and GluR2 mRNA levels was detected in the different brain regions of freshwater (FW) lungfish (Figure 2). In particular, anterior brain regions (Figure 2a) supplied intermediate (0.5 < OD < 0.75) GluR1 and GluR2 mRNA levels in the lateral septum (Sl) and in the dorsal pallium (Dp), respectively, while lower expression capacities (OD < 0.5) for both subtypes characterized other brain regions such as lateral pallium (Lp), dorsal portion of medial pallium (Pd) and medial subpallium (Sm). In posterior regions (Figure 2b), high (OD > 0.75) GluR1 mRNA levels characterized the dorsal part of the hypothalamus (Hyd) along with intermediate levels being reported for the pars intermedia of reticular nucleus (Ri), whereas low OD signals were typical of the optic tectum (Te), dorsal thalamus (Thd) and corpus cerebelli (Cc). Interestingly of all the posterior brain areas, this last romboencephalic region was the only site supplying high GluR2 expression levels.

### GluR1 and GluR2 mRNA levels during normoxic and hypoxic aestivation

**Figure 3:**
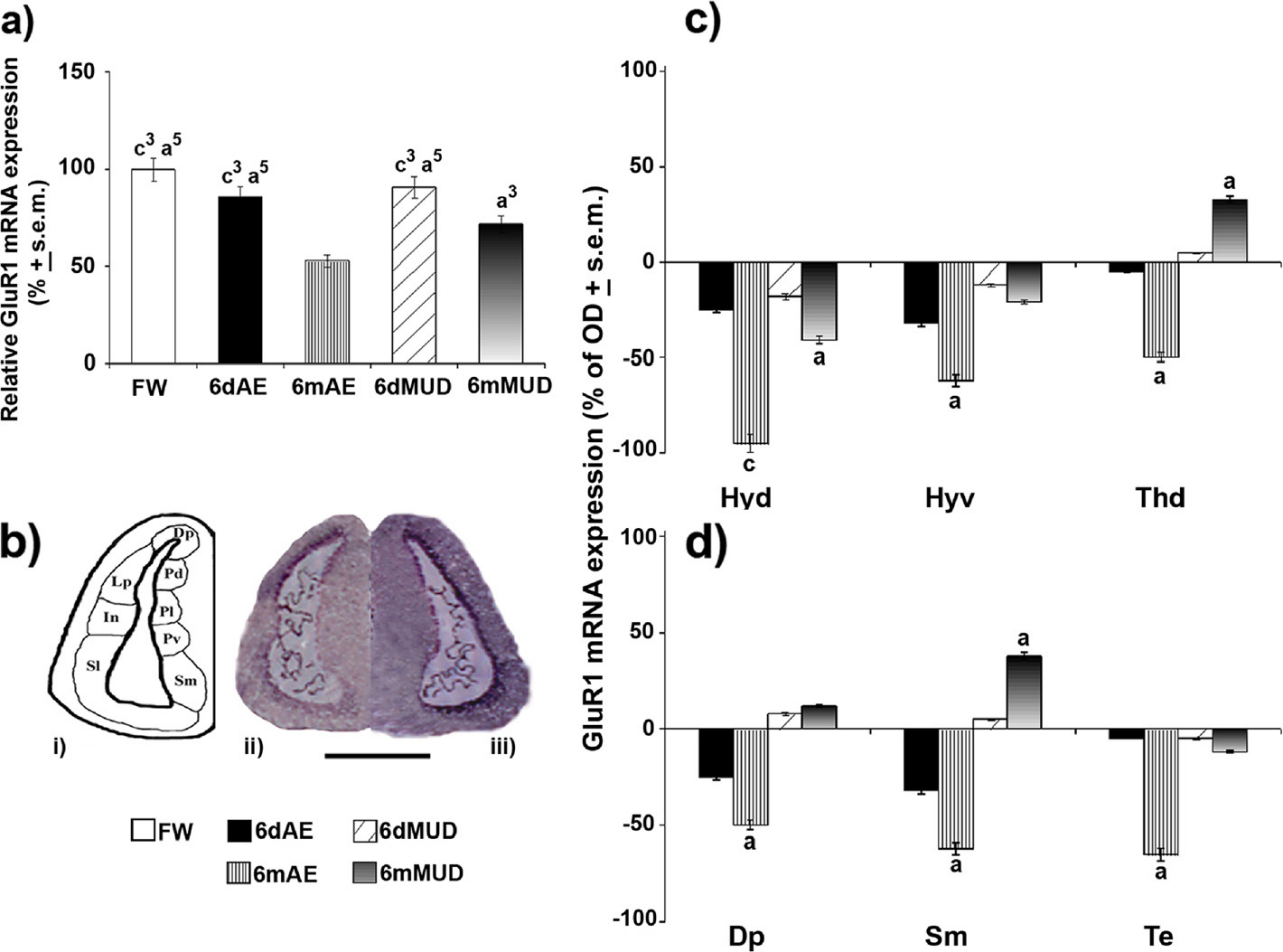
**GluR1 mRNA expression during normoxic and hypoxic aestivation.** mRNA levels were evaluated in whole brain of FW (white^1^), 6dAE (black^2^), 6mAE (vertical striped^3^), 6dMUD (diagonal striped^4^) and 6mMUD (grey^5^) animals by qPCR. **(a)** The results are expressed as a proportion of the highest value after normalization with respect to β-actin expression levels and represent the means ± s.e.m. of three independent biological replicates. GluR1 *in situ* hybridization pattern was handled in the different brain nuclei of diencephalic **(c)** and extradiencephalic **(d)** areas of all aestivating conditions (expressed as % of FW). In particular, an evident reduction of mRNA levels was typical of 6mAE (*ii*) with respect to 6mMUD (*iii*) lungfish in telencephalic regions **(b)**, of which a schematic representation was reported (*i*). Statistical analysis: ANOVA followed by Student’s *t* test for qPCR data and by Newman Keul’s test for *in situ* hybridization (^a^*p* < 0.05, ^b^*p* < 0.01, ^c^*p* < 0.001). Scale bar: 600 μm. For abbreviations check list.

The discrimination of GluR1/2 expression capacities throughout the various brain regions constituted an essential step towards the recognition of these AMPAergic subtypes as major targets of aestivating conditions. qPCR analysis carried out on the whole brain of the different experimental conditions exhibited a very high three-fold (*p* < 0.001) reduction of GluR1 mRNA levels in lungfish maintained under a long normoxic aestivation (6mAE) with respect to FW, 6dAE and 6dMUD states, along with moderate (*p* < 0.05) reductions with respect to 6mMUD animals (Figure 3a). Moreover, this latter condition displayed a moderately (*p* < 0.05) significant decrease when it was compared to FW, 6dAE and 6dMUD states (Figure 3a). Contextually to this trend, *in situ* hybridization data (Figure 3b-d) supported a preferential brain regional-dependent down-regulation of GluR1 mRNA expression for lungfish maintained under long normoxic aestivating conditions (Figure 3b*ii*) with respect to hypoxic 6mMUD (Figure 3b*iii*). In particular a very high reduction of GluR1 transcripts occurred in mostly diencephalic areas such as Hyd of 6mAE animals when compared to FW (Figure 3c). At the same time, moderate reductions were typical of the ventral part of hypothalamus (Hyv) and Thd in 6mAE conditions (Figure 3c). During the long hypoxic aestivating state (6mMUD), a moderate down- and up-regulation of GluR1 levels characterized Hyd and Thd, respectively, when compared to that of FW conditions. It is worthy to note that when GluR1 variations were estimated for extra-diencephalic areas, 6mAE animals still continued to exhibit a general down-regulatory pattern, even though of moderate entity, as reported in some telencephalic regions such as Dp and Sm as well as in Te (Figure 3d). Conversely, a moderate increase of GluR1 transcripts was predominantly typical of ventral telencephalic brain areas, such as Sm, when lungfish were maintained under a 6mMUD state with respect to FW conditions (Figure 3d).

**Figure 4:**
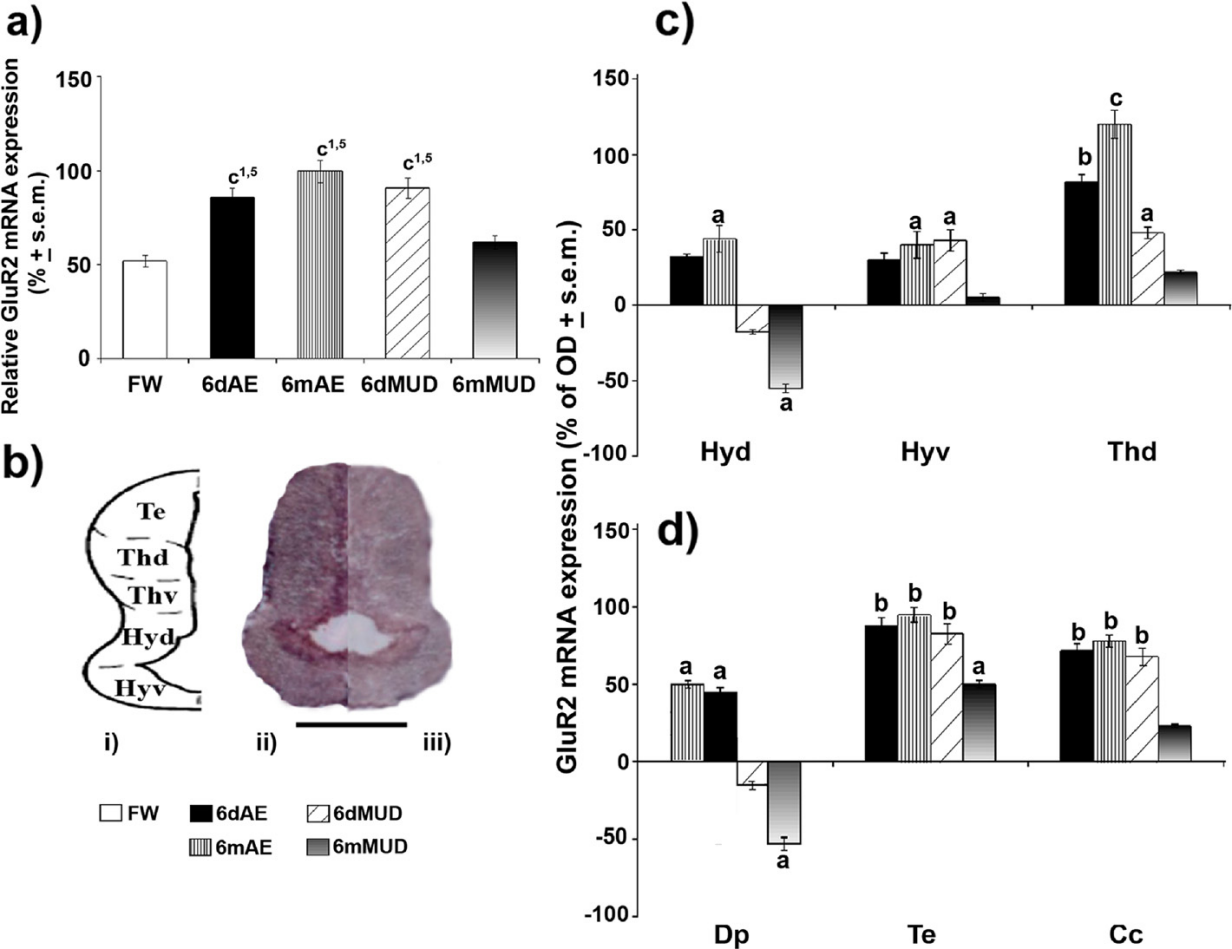
**GluR2 mRNA expression during normoxic and hypoxic aestivation.** mRNA levels were evaluated in whole brain of FW (white^1^), 6dAE (black^2^), 6mAE (vertical striped^3^), 6dMUD (diagonal striped^4^) and 6mMUD (grey^5^) animals by qPCR. **(a)** The results are expressed as a proportion of the highest value after normalization with respect to β-actin levels and represent the means ± s.e.m. of three independent biological replicates. GluR2 *in situ* hybridization pattern was handled in the different brain nuclei of diencephalic **(c)** and extradiencephalic **(d)** areas of all aestivating conditions (expressed as % of FW). In particular, an evident increase of mRNA levels was detected in 6mAE (*ii*) with respect to 6mMUD (*iii*) lungfish at diencephalic level **(b)**, of which a schematic representation was reported (*i*). Statistical analysis: ANOVA followed by Student’s *t* test for qPCR data and by Newman Keul’s test for *in situ* hybridization (^a^*p* < 0.05, ^b^*p* < 0.01, ^c^*p* < 0.001). Scale bar: 600 μm. For abbreviations check list.

As far as GluR2 expressing neurons are concerned, they resulted to be active at a rather short aestivating state, thus accounting for up-regulated mRNA levels as early as 6 days for both normoxic and hypoxic conditions. Indeed, qPCR analysis demonstrated a three-fold (*p* < 0.001) increase of GluR2 levels in both 6dAE and 6dMUD conditions with respect to FW and 6mMUD animals. The rising trend of GluR2 continued to be also maintained for 6mAE animals whereas in the case of 6mMUD animals the expression capacity of this AMPAergic subtype was strongly reduced to control values (Figure 4a). The transcriptional levels of GluR2 (Figure 4b-d) resulted to be preferentially concentrated in diencephalic regions of 6mAE (Figure 4b*ii*) with respect to 6mMUD animals (Figure 4b*iii*). Indeed, evident up-regulations were reported for Thd in 6mAE (*p* < 0.001) and 6dAE (*p* < 0.01), while a moderate up-regulation appeared to characterize Hyd and Hyv only following long normoxic conditions (Figure 4c). However, hypoxic aestivating states did not appear to promote a clear-cut direction for diencephalic sites on the account of a heterogeneous expression pattern being featured by all conditions especially in the case of moderate increases in Thd and Hyv of 6dMUD state as compared to a moderate down-regulation for Hyd of 6mMUD conditions (Figure 4c). Conversely, an up-regulatory type of effect characterized above all extra-diencephalic areas, since aside the moderate down-regulated patterns in Dp of 6mMUD group with respect to FW state (Figure 4d), high GluR2 mRNA levels were instead reported for Te and Cc of 6dAE, 6dMUD and 6mAE groups along with moderate up-regulations characterized Dp of short and long normoxic conditions (Figure 4d). In addition, neurons expressing this AMPAergic receptor subtype in 6mMUD animals appeared to be also typical of mesencephalic areas as shown by a moderate GluR2 mRNA up-regulation in Te.

### mRNA expression of protective factors and TUNEL reaction during normoxic and hypoxic conditions

In a same manner as for AMPA receptor subtypes, even the partial coding sequences for both HSP70 [GenBank: HQ993059] and HSP27 [GenBank: JF262201] as well as for HIF-1α [GenBank: 1457856; *work in progress*] in *Protopterus annectens* were obtained by using primers designed on rat (HSPs) and on zebrafish (HIF-1α) heterologs (Figure 1a). In *Protopterus annectens* HSP70 (Figure 1d) partial sequence showed a high nucleotide identity (>75%) with the heterologs of *Rattus norvegicus* [GenBank: L16764] and fish *Thalassoma pavo* [GenBank: EF392849] and *Danio rerio* [GenBank: AF006007]; HSP27 (Figure 1e) with the heterologs of *Rattus norvegicus* [GenBank: M86389] and *Salmo salar* [GenBank: BT048553]; HIF-1α (Figure 1f) with heterologs of *Rattus norvegicus* [GenBank: AF057308] and fish *Cyprinus carpio* [GenBank: EU144225] and *Danio rerio* [GenBank: AF525743].

**Figure 5:**
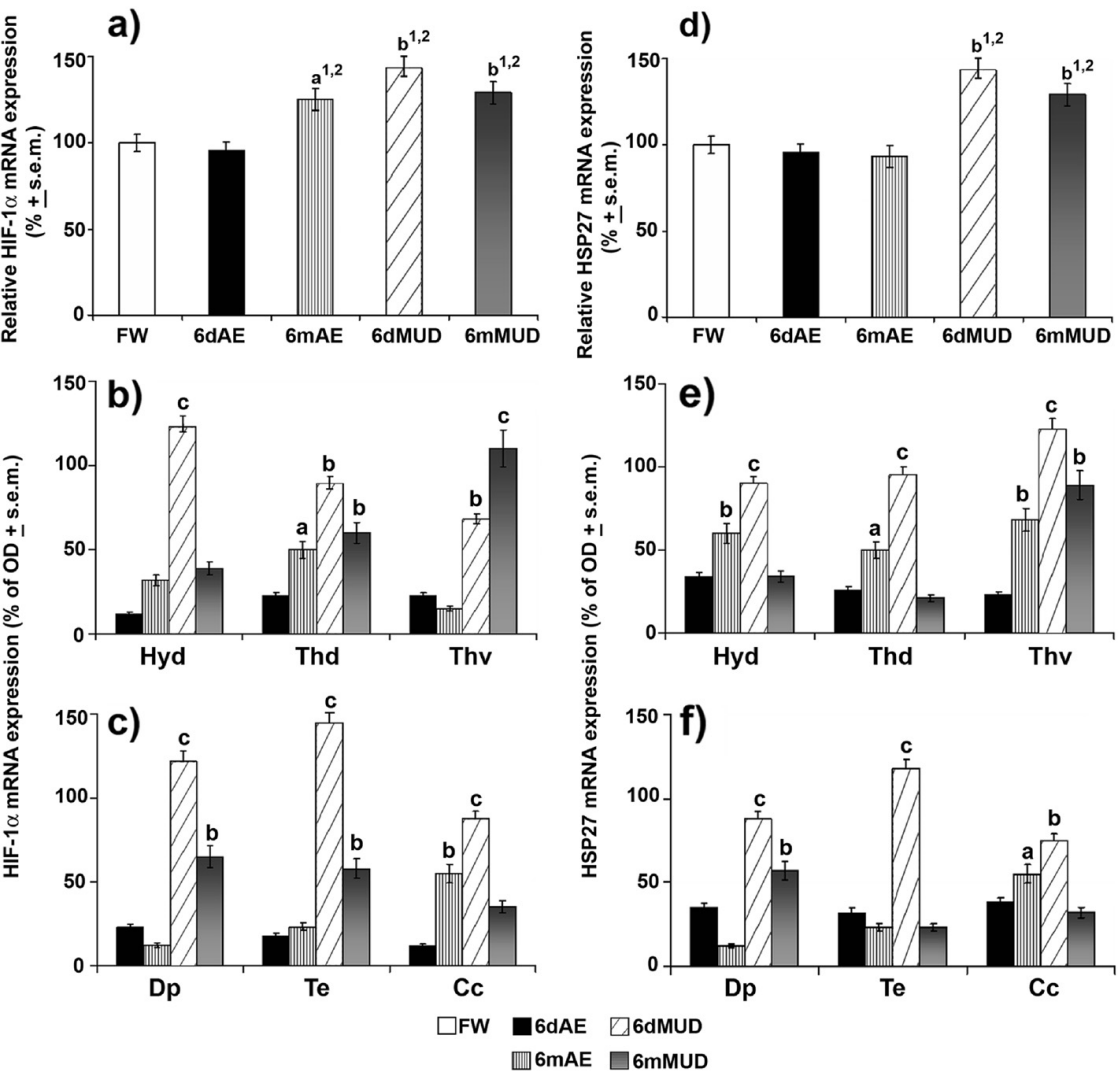
**HIF‒1α and HSP27 mRNA expression during normoxic and hypoxic aestivation.** mRNA levels were evaluated in whole brain of FW (white^1^), 6dAE (black^2^), 6mAE (vertical striped^3^), 6dMUD (diagonal striped^4^) and 6mMUD (grey^5^) animals by qPCR. **(a,d)** The results are expressed as a proportion of the highest value after normalization with respect to β-actin levels and represent the means ± s.e.m. of three independent biological replicates. HIF‒1α and HSP27 *in situ* hybridization pattern was handled in the different brain nuclei of diencephalic **(b,e)** and extradiencephalic **(c,f)** areas of all aestivating conditions (expressed as % of FW). Statistical analysis: ANOVA followed by Student’s *t* test for qPCR data and by Newman Keul’s test for *in situ* hybridization (^a^*p* < 0.05, ^b^*p* < 0.01, ^c^*p* < 0.001). For abbreviations check list.

**Figure 6:**
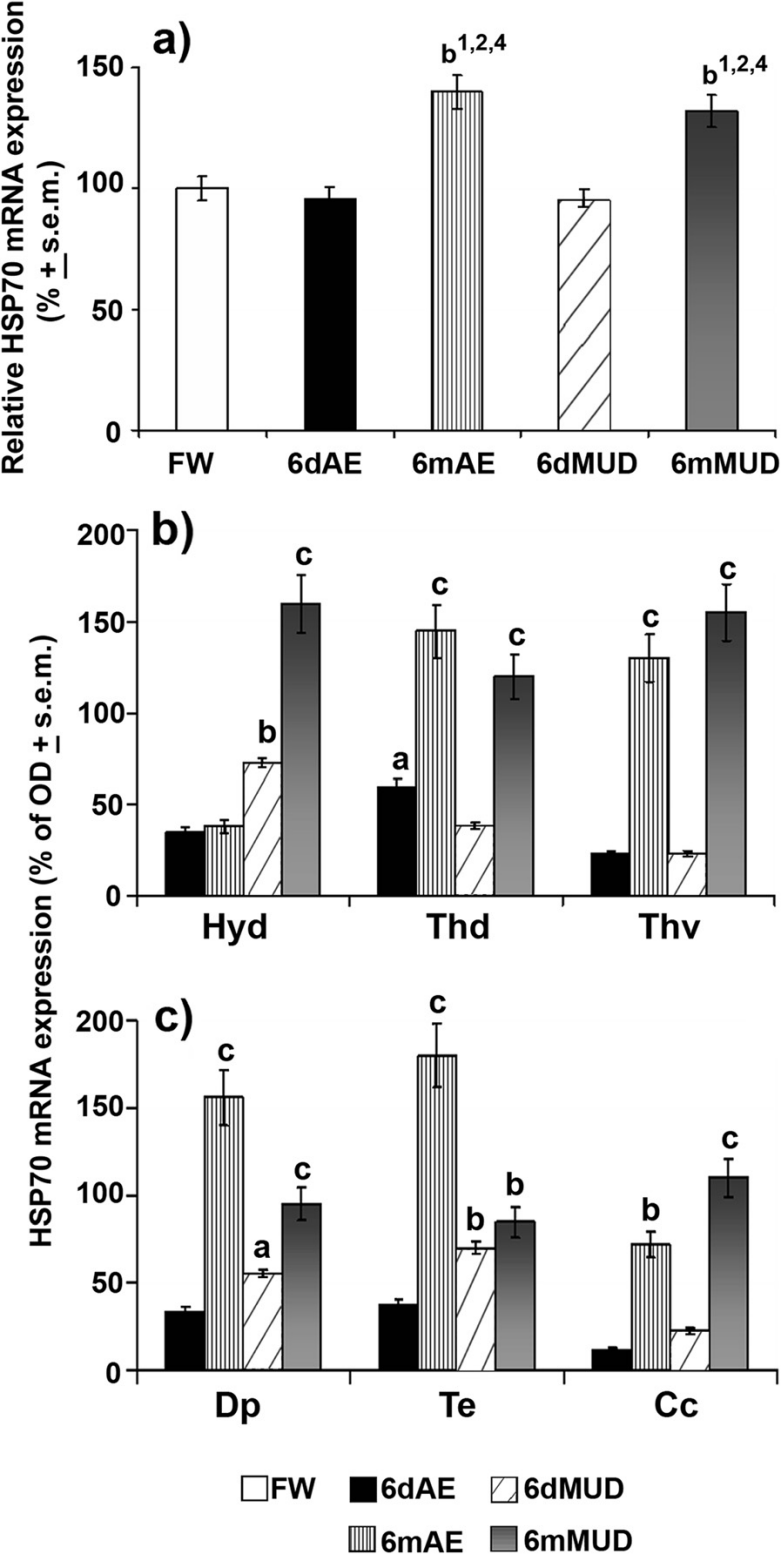
**HSP70 mRNA expression during normoxic and hypoxic aestivation.** mRNA levels were evaluated in whole brain of FW (white^1^), 6dAE (black^2^), 6mAE (vertical striped^3^), 6dMUD (diagonal striped^4^) and 6mMUD (grey^5^) animals by qPCR. **(a)** The results are expressed as a proportion of the highest value after normalization with respect to β-actin levels and represent the means ± s.e.m. of three independent biological replicates. HSP70 *in situ* hybridization pattern was handled in the different brain nuclei of diencephalic **(b)** and extradiencephalic **(c)** areas of all aestivating conditions (expressed as % of FW). Statistical analysis: ANOVA followed by Student’s *t* test for qPCR data and by Newman Keul’s test for *in situ* hybridization (^a^*p* < 0.05, ^b^*p* < 0.01, ^c^*p* < 0.001). For abbreviations check list.

Subsequently, qPCR and *in situ* hybridization analyses supplied a differentiated mRNA expression pattern of these neuroprotective factors during both normoxic and hypoxic conditions. Such protective elements showed a preferential type of activity not only for short hypoxic states (HIF-1α, HSP27) but also for long hypoxic and normoxic states (HSP70). Indeed, HIF-1α and HSP27 supplied an overlapping expression pattern and precisely a high two-fold (*p* < 0.01) increase being typical of short (6dMUD) and long (6mMUD) conditions with respect to both FW and 6dAE groups (Figure 5a,d). At the same time, HIF-1α also exhibited a moderate transcriptional increase in 6mAE animals with respect to these same conditions. In the case of HSP70, this chaperone appeared to characterize exclusively long aestivating conditions, as demonstrated by two-fold increases in 6mAE and 6mMUD with respect to all experimental conditions (Figure 6a).

From *in situ* hybridization evaluations, it seemed that the specific distribution pattern of neurons expressing GluR1 and GluR2 tightly overlapped those expressing HIF-1α and HSP27 as well as HSP70 under the above aestivating conditions. The different neuronal fields of diencephalic and extra-diencephalic areas turned out to be the main expression sites of HIF-1α transcripts during mainly short and long hypoxic conditions, as displayed by very high mRNA transcript levels of this transcriptional factor in Hyd (+125%), Dp (+120%), Te (+132%) and Cc (+92%) along with high levels in Thv (+70%) and Thd (+87%) of 6dMUD animals with respect to FW state (Figure 5b,c). Some of these same brain areas, such as Thv, continued to express very high levels of HIF-1α mRNA (+115%) under long hypoxic aestivating condition (Figure 5b), along with up-regulations being typical of Thd (+62%), Dp (+60%) and Te (+58%). In the case of normoxic aestivating state, 6mAE lungfish appeared to be characterized by significant HIF-1α increases (Figure 5b,c) in Cc (+56%; *p* < 0.01) and Thd (+46%, *p* < 0.05). At the same time, early hypoxic conditions also accounted for very high HSP27 mRNA levels in these same brain areas (Figure 5e,f) and namely Thv (+125%), Te (+112%), Hyd (+95%), Thd (+98%) and Dp (+90%) along with increases in cerebellar areas (Cc, +75%). Under a long hypoxic state (6mMUD), HSP27 turned out to be expressed in a still significant fashion as reported by its high level in Thv (+80%) and Dp (+58%), while during the long normoxic aestivation (6mAE) an evident up-regulation characterized Hyd (+56%) and Thv (+64%; Figure 5e) and moderate increases were detected in Thd (+46%) and Cc (+48%; Figure 5f).

**Figure 7:**
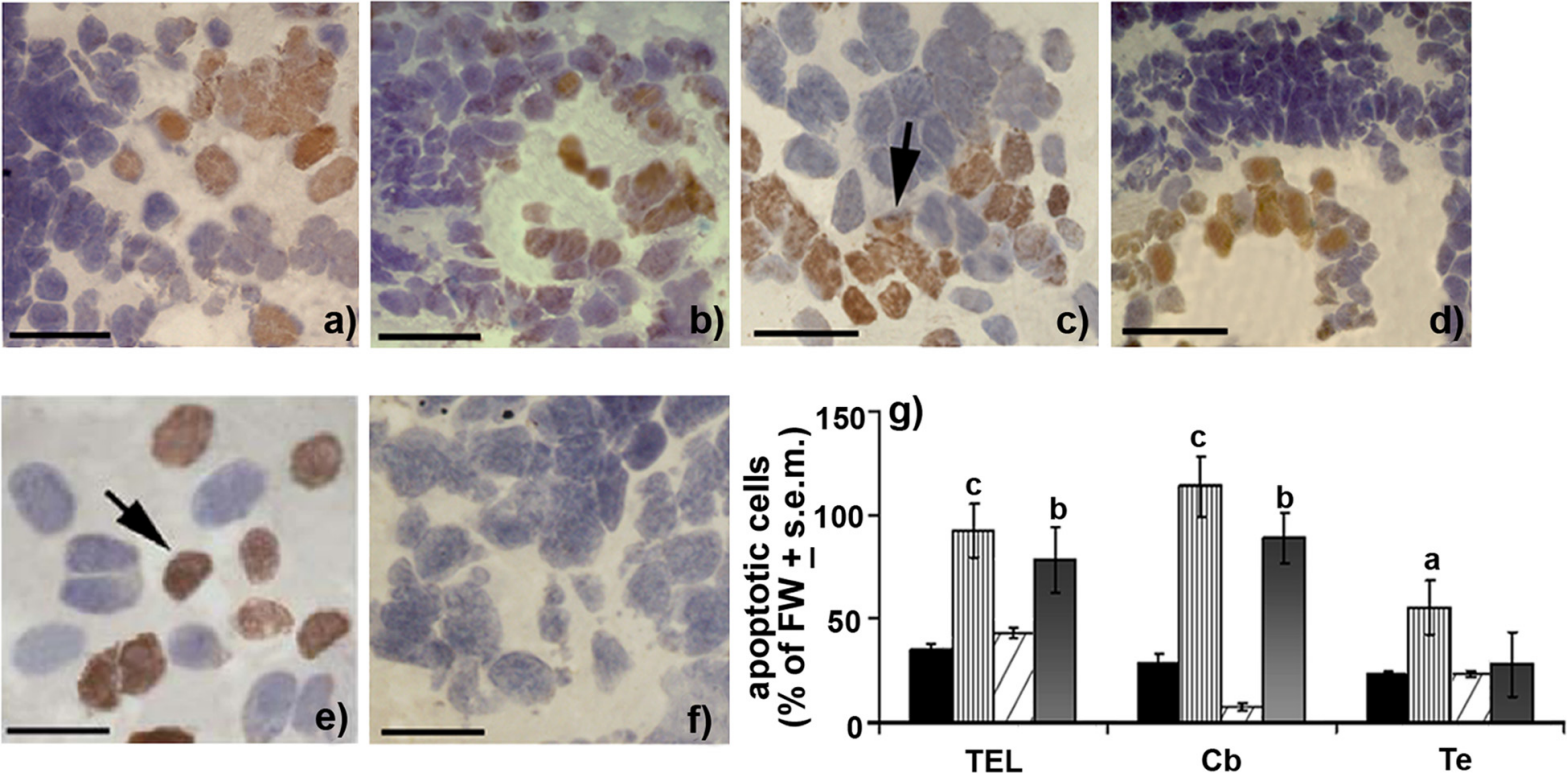
**Apoptotic events during normoxic and hypoxic aestivation.** TUNEL signals were evaluated in 6dAE (black), 6mAE (vertical striped), 6dMUD (diagonal striped) and 6mMUD (grey) states. At 6mAE state, an evident amount of neuronal fields containing apoptotic cells (TUNEL method, black arrow) was detected in TEL **(a)**, Cb **(c)** and Te **(e)** with respect to the representative TUNEL control **(f)**. In the case of 6mMUD condition, TEL **(b)** and Cb **(d)** continued to supply an evident TUNEL reaction. Variations in apoptotic cell content **(g)** were reported as % of apoptotic cells ± s.e.m. with respect to FW and analyzed by ANOVA and Newman Keul’s test (^a^*p* < 0.05, ^b^*p* < 0.01, ^c^*p*<0.001). Scale bars (a-d) = 80 μm; (e,f) = 40 μm. For abbreviations check list.

As far as mRNA levels of HSP70, which has been shown to be indicative of a long-term hypoxia-related protective measure are concerned, this transcript was expressed under prevalently long normoxic and hypoxic aestivating states with respect to other experimental conditions (Figure 6a). *In situ* hybridization data showed that very high expression levels of this protective factor were specific for diencephalic areas (Figure 6b), such as Thd (+145%) and Thv (+128%) under long normoxic aestivating conditions, with a similar pattern being also obtained for long hypoxic aestivating conditions in Thd (+120%), Thv (+155%) and Hyd (+158%). On the other hand, a significant increase of HSP70 was registered for Hyd (+72%) of 6dMUD animals and for Thd (+54%) of 6dAE (Figure 6b). In line with this up-regulating trend, the mRNA expression pattern of such a protective factor appeared to be also highly increased in Dp (+152%) and Te (+180%) during long normoxic aestivating conditions along with a significant increase for Cc (+70%; Figure 6c). Similarly, long hypoxic conditions continued to induce a very high up-regulation of HSP70 transcripts, this time in Dp (+95%) and Cc (+108%), together with increases in Te (+74%), while the short hypoxic state accounted for significant up-regulation of mRNA levels in Dp (+55%) and Te (+70%; Figure 6c). Surprisingly, the expression pattern of this protective factor seemed to overlap apoptotic events in extra-diencephalic brain regions under long aestivation conditions (Figure 7g). This was particularly evident for the very great number of TUNEL positive neurons for the entire TEL (+95%; Figure 7a) and Cb (+110%; Figure 7c) under a long normoxic state (6mAE), along with a moderate reaction being also detected in Te (+55%; Figure 7e) for this same aestivating state with respect to FW (Figure 7f). At the same time, an evident TUNEL reaction characterized TEL (+78%; Figure 7b) and Cb (+88%, Figure 7d) when lungfishes were exposed to long hypoxic aestivation conditions (6mMUD).

## Discussion

In this work for the first time, altered transcriptional levels of both AMPAergic receptor subtypes mRNAs occurring concomitantly to elevated expression levels of selective neuroprotective factors during the different aestivating conditions tend to underlie a distinct protection program operating in a similar fashion to those reported for some ischemic disorders [21,25]. This feature was basically established through the detection of the specific silent neurons expressing GluR1 and/or GluR2 mRNAs in well-known motor-related brain regions such as Cb and Te of the lungfish, so suggesting that compensatory pathways under hypoxic conditions might be activated [26]. In such a context it appears that the switching over between mRNA expression of these two subtypes under either brief or long aestivating states may turn out to be a pivotal condition for the functional recovery of motor activities at arousal, which are controlled by these areas very likely via the action of AMPA receptors permeable to Ca^2+^ lacking GluR2 [27,28]. In addition, these same brain areas featured enhanced expression levels of the neuroprotective factors HIF-1α, HSP27 and HSP70, which were comparable to those of other aestivating species such as the turtle *Trachemys scripta*[1] and the lizard *Tupinambis merianae*[29] that display a notable resistance to not only ammonium toxicity [6] but also to oxidative state deficits [30,31]. In line with this condition, it is very probable that the specific molecular properties of motor brain sites seem to constitute a key target of oxygen-dependent nitrogen metabolism of aestivating species [32] and as a consequence these tend to operate in a very similar manner to hypoxia-related neurodegenerative disorders like Alzheimer’s Disease in mammals [33].

Curiously, both AMPA receptor subtypes were not expressed in the same direction especially during reduced oxygen availability of aestivation in mud and this seems to point to the induction of GluR1/2 as a key step responsible for the consolidation of silent synaptic processes, at least during sleeping states [34,35]. Such a feature was established via the identification of partial nucleotide fragments codifying for both AMPA receptor subtypes in *Protopterus annectens*, which subsequently enabled us to display a putative high degree of similarity to that of not only fish but also to those of amphibians [36], birds [37] and mammals [38]. Indeed, high GluR2 mRNA levels above all in thalamic and mesencephalic regions may constitute ideal situations at least for the maintenance of long sleeping-like states of lungfish, this time not only during normoxic aestivating conditions but above all during early aestivating states. These conditions are very likely due to high GluR2 mRNA levels being tightly related to silent neuron type of events that consist in lysosomial degradative pathways promoting AMPA internalization processes [27]. Hence this phenomenon, like that of NREM in mammals, appears to heavily rely on low GluR1 levels detected during the long normoxic aestivating conditions, very probably through their inactivation by protein kinase C II-related processes [35,39]. The fact that both silent subtypes are involved with sleeping events of our lungfish tends to indicate a gradual insertion of AMPA receptor-dependent excitatory synaptic functions being essential for numerous neuronal activities such as the ionic channel pharmacological properties of the different vertebrates [36,40] and above all initial contact-related motor functions that are typical of zebrafish embryos [41].

The results of TUNEL analysis and the concomitant increased HSP70 levels seem to suggest that a specific homeostatic neuronal program involving neurogenesis and apoptotic events are characterizing the different aestivating conditions of *Protopterus annectens.* In particular, specific motor controlling neuronal fields during long aestivating conditions (6mAE and 6mMUD) exhibited dense apoptotic reactions similar to those obtained for other vertebrates such as amphibians [42] and mammals [43]. It is thus tempting to propose that the lungfish brain during aestivation executes a precise balance between cell death and neurogenesis especially in those areas, which are required to maintain in equilibrium an accurate number of cells in a regenerative state [44], probably via HSP70-dependent suppression of necrosis and caspase-independent apoptotic pathways [45]. Interestingly, HSP70 has been recognized as a “conformational repair agent” and so its elevated levels during long aestivating states like that of brain hyperthermic stress may prove to be important for correcting stress-induced damage to synaptic proteins and guiding the structural assembly at postsynaptic density [46]. Moreover, the preservation of neurosignaling activities during long aestivation requires the activation of the pro-survival factor HSP70 in motor-related areas similarly to protective mechanisms detected in mammals during cerebral ischemia-dependent disorders [47].

It is worthy to note that during hypoxic aestivating conditions AMPA receptor transcriptional levels were increased, as demonstrated by significant mRNA levels of GluR1 and GluR2 during 6mMUD and 6dMUD states, respectively. Similar receptor variations have been reported during short-term hypoxia for rat hippocampal AMPA receptor complex [48], while under prolonged hypoxic conditions an increase of AMPA current is not observed, indicating its suppression during prolonged oxygen deprivation. Consequently, the increase of the two silent neurons during brief hypoxia may contribute to an early reorganization of synapses, including the promotion of AMPA receptor-mediated effects at previously silent synapses and increased synthesis of excitatory receptor subtypes [49,50]. Subsequently the blockade of AMPAergic currents, through the recruitment of AMPA receptors containing GluR2, may promote long-term neuronal survival and tolerance to seizure susceptibility in those brain areas of the lungfish linked with the recovery of motor and visual functions at arousal. These plasticity events appear to exhibit a similar trend to that observed in the anoxic turtle brain [51], suggesting that conserved protective systems may be adopted during vertebrate evolution, in order to assure rapid and appropriate responses to various oxygen tensions. The early increase of GluR2 mRNA levels in 6dMUD condition, promptly replaced by a reduction of this transcript during 6mMUD state, may be considered as a pre-conditioning factor against hypoxia-related events, which appears to strongly require the rapid induction of HIF-1α and HSP27. Such an activity is in line with recent evidences showing that stress-related proteins may display a different temporal activation during hypoxia, with the protective factor (HSP27) representing an early switch against hypoxia-related conditions in newborn mammals [52,53]. Furthermore, the harsh environmental conditions guiding the lungfish into a hypometabolic state promotes the activation of the early protective factors that causes the arrest of growth/developmental events as well as of apoptotic neuronal processes [43]. Thus, neuronal death occurring after a short hypoxic aestivation in our lungfish may account for a rapid activation of HIF-1α, which in turn promotes the recruitment of inducible HSP27. This small chaperone by activating a protective pathway through the inhibition of both caspase-dependent and -independent mechanisms [22] prevents neuronal damages against subsequent ischemic insults in a similar manner to that reported for rat retinal ischemia [54].

## Conclusions

These results underlie the crucial role of the expression of the AMPAergic silent neurons (GluR1/R2) during aestivation of *Protopterus annectens* and highlight new adaptive strategies operating in key brain regions of this lungfish in relation to oxygen availability. During vertebrate evolution, the homoeostatic balance between an active or silent synaptic state resulted to be a determinant factor for the entering into a torpor condition, probably via the regulation of GluR1 and GluR2 phosphorylative processes [55]. GluR2 seems to represent an early neuronal marker of cellular oxidative imbalance, a condition by which this AMPA receptor subtype concomitantly to HIF-1α/HSP27 complex elicits long-term protection during aestivation, in a similar fashion to that reported during mammalian ischemic events [56]. In particular, long-term expression variations of silent neurons that are tightly related to HSP70-dependent synaptic stabilization may bring us closer to elucidate the type of neuronal mechanism(s) operating during hypoxia-related dysfunctions [57]. We are still at the beginning, but the application of pharmacological and molecular approaches for the characterization of distinct AMPA receptor membrane domains as well as their interaction with other hypoxia-related factors, such as vascular endothelial growth factor [58] may represent a valuable therapeutic tool for hypoxia-related disorders such as ischemia.

## Methods

### *Maintenance of specimens*

The study was performed on the African lungfish *Protopterus annectens* (body weight 140–190 g). Specimens (n = 60) were collected from Central Africa and imported through a local fish farm in Singapore (Qian Hu Fish Farm Trading Co, Singapore). In this work animal sex was not taken into consideration since its identification is not possible due to the lack of distinct external features. All lungfish were acclimatized to laboratory conditions for at least 1 month in order to eliminate any non-specific effects deriving from stressful ambient states before the beginning of experimental trials. They were maintained in plastic aquaria filled with dechlorinated tap water (pH 7.1–7.2), containing 0.71 mM Na^+^, 0.32 mM K^+^, 0.72 mM Ca^++^, 0.06 mM Mg^++^, 2.2 mM Cl^−^ and 0.2 mM HCO_3_^−^, at 25°C under 12-h light/12-h dark photoperiod until use and water was changed daily. During the adaptation period, animals were fed frozen bloodworms up to a period of 96 h before experimental session in which food was withdrawn. Experimental protocols of this study were approved by the National University of Singapore Institutional Animal Care and Use Committee (IACUC Permits 813/05, S06/06 A and 035/09).

### *Aestivation*

Lungfishes were exposed to two different type of aestivation and precisely in air (normoxia) and mud (hypoxia) conditions, according to previous studies [6,32]. For this purpose some fish were induced to aestivate in air at 25–30°C individually in plastic tanks (29 cm x 19 cm x 17.5 cm, length x width x height) containing a small volume (15 mL) of dechlorinated tap water. Water dried up in approximately 6 days and, during this time, animals formed a mucus cocoon that enveloped the entire body. At the same time, for the handling of mud aestivation dried mud collected from the bottom of freshwater ponds was purchased from Hua Hing Trading Co (Singapore). The dried mud was soaked in dechlorinated tap water for at least 2 days, and mixed into a thick paste (approximately 30% water content) by hand. Artificial muddy substrata (19 kg dry mass) with a minimum depth of 15 cm were made in similar plastic tanks used for air aestivation. Fish (one per tank) were allowed to bury at liberty into mud, which took 2–12 h. A small amount of water (approximately 100 mL) was evenly spread on to the surface of the mud every 4–5 days to prevent the surface mud from drying up and cracking. In addition, there was a small air passage, which connected the point of entry from the mud surface to the aestivating fish. However, in all cases, the anterior end of the fish was observed to be orientated away from the air passage. Subsequently animals (n = 12 for each condition) were sacrificed during the different aestivating states: at the induction phase after 6 days of aerial normoxic (6dAE) or mud hypoxic (6dMUD) aestivation, that may be considered as short entering states into aestivation [6]; after 6 months of aerial normoxic (6mAE) or mud hypoxic (6mMUD) aestivation defined as the long maintenance phase of such a state. Other fish (n = 12), maintained in freshwater (FW) conditions were identified as controls and placed in dechlorinated tap water for the same duration that was used for all aestivating lungfish. Afterwards all animals were sacrificed and their dorsal skull was opened in order to extract their brain, which was stored at −80°C and −20°C for future analyses. For the different experimental phases described in this study, the number of animals for each conditions (n = 4) was assessed according to Mead’s resource equation, which is in line with our previous published works [4].

### *qPCR analysis*

For this study, we directed our attention to the expression pattern of the AMPAergic silent neurons (GluR1 and GluR2) along with some neuroprotective factors and namely HIF-1α, HSP70 and HSP27 during normoxic and hypoxic aestivation of the lungfish. For this aim, brains of *Protopterus annectens* belonging to 6dAE (n = 4), 6dMUD (n = 4), 6mAE (n = 4), 6mMUD (n = 4) and FW (n = 4) groups, as described above, were quickly removed from the skull and stored at −80°C. Total RNA was extracted from whole brain of each experimental group using TRI reagent (Sigma, Italy) and the quality of RNA samples was assured by measuring optical density (OD, 260/280) absorption ratio (range 1.62 – 2.1), while their integrity was verified by the detection of 18 S and 28 S bands after agarose gel electrophoresis. Total RNA (2 μg) of each sample was used to synthesize cDNA according to indications of the High Capacity cDNA Reverse Transcription Kit (Applied Biosistem, Italy). PCR using Taq Polymerase (Promega, Italy) was handled for both GluR1 and GluR2, along with HIF-1α, HSP70 and HSP27 by using primers pairs reported in Figure 1 and putative partial cds sequences were firstly obtained for lungfish and submitted to GenBank database. Quantitative real-time PCR (qPCR) was performed on a Biorad Miniopticon (Biorad, Italy) single color thermocycler and experimental procedures were established on the basis of previously reported guidelines [59]. Amplification reaction was prepared in a final volume of 25 μL by adding 12.5 μL of SYBR-Green Supermix, 0.3 μM of primers for target genes, 0.1 μM of primers for β-actin and 10 ng of each cDNA. All reactions were run in triplicate according to the following cycling parameters: one cycle at 94°C for 3 min, 40 cycles of denaturation at 94°C for 10 s and annealing-extension at 57°C for 30 s. After the reaction, the existence of a unique PCR product ranging from 150 to 200 bp was confirmed via melting curve analysis [60], obtained by an increase of 0.5°C every 10 s from 57°C to 95°C. After checking independent trials of several reference genes, the β-actin reported the most reproducible results across the various cDNAs and so it was used as normalization gene. PCR amplification efficiency for each primer pair was calculated on the basis of the slope of the standard curve, which in most cases was close to −3.4, indicating maximal PCR amplification efficiency with a high correlation coefficient of amplification accuracy within the range 0.990–0.998. Results of qPCR were analyzed using Opticon Monitor qPCR detection system (Biorad), with a program that permits the analysis of the reaction kinetic. *C*_q_ values were obtained with Genex software (BIORAD) and data were analyzed according to the method of 2^−ΔΔCT^[61] on the basis of gene expression levels calculated from three biological repeats. Data obtained for all groups were reported as a proportion of the highest value after normalization and statistical differences were estimated by one-way ANOVA followed by a *post hoc* Student’s *t* test when there was a significant p-value < 0.05.

### *In situ hybridization analysis*

The specific neuronal fields responsible for the mRNA encoding of GluR1 and GluR2 subtypes as well as for HIF-1α and HSP27/70 in distinct brain nuclei was investigated by performing *in situ* hybridization in the different aestivating groups. For this purpose, antisense and sense probes were designed on the basis of the partial sequences obtained in *Protopterus annectens* for all gene targets considered in this study and labeled by 3′-tailing using digoxigenin-11-dUTP (Roche, Italy), as previously reported [62]. Briefly, 100 ng of antisense probe was added to brain sections (14 μm) of the lungfish belonging to 6dAE, 6dMUD, 6mAE, 6mMUD and FW conditions (n = 4/group). Afterwards the probe was left overnight at 50°C in a humidified chamber. Non-specific hybridization was handled on slides incubated with sense probe. For immunological detection anti-DIG alkaline phosphatase antibody (1:100, Roche, Milan) was added for 2 h at room temperature, preceded by 45 min permanence on Blocking Reagent (1%, Roche, Milan). Neuronal hybridization signals were observed at a bright-field Dialux EB 20 microscope (Leitz, Stuttgart, Germany; 16X magnification; 25°C and Leica Imaging software for image acquisition). Transcription differences for anterior, intermediate and posterior brain areas of both sections were evaluated by using a Macintosh computer-assisted image analyzer system running Image software of National Institutes of Health (Scion Image 2.0) and an internal standard for OD calibration. Before commencing with the calibration of mean OD value for all sections an estimation of the background level, which was processed automatically by Scion Image program, was elaborated and included for all final calculations.

### *Neuronal apoptosis assay*

The determination of apoptosis was also handled on lungfish brains that belonged to 6dAE (n = 4), 6dMUD (n = 4), 6mAE (n = 4) and 6mMUD (n = 4) with respect to FW (n = 4) by using a kit for immunohistochemical detection and quantification of apoptosis based on TUNEL technology (*In situ* Cell Death Detection Kit POD, Roche-Italy). Briefly, fixed brain sections (16 μm) were incubated in TUNEL reaction mix (label ± terminal transferase solution) for 60 min at 37°C. Then a solution of sheep anti-fluorescein antibody conjugated with horseradish peroxidase (POD) was added to brain sections for 45 min at 37°C and the colorimetric reaction was obtained by adding diaminobenzadine (DAB) substrate (20 min at 25°C). Hematoxylin counterstained slides were cover-slipped with Di-N-butyl-Phthalate in Xylene (DPX) mounting medium for light microscopy observation. Brown stained TUNEL-positive apoptotic cells were observed in a bright-field Dialux EB 20 microscope (Leitz, Stuttgart, Germany; 16-40X magnification; 25°C and Leica Imaging software for image acquisition). Because of the same negative results, only one representative control was illustrated and compared to the brain areas of all groups. For quantitative analysis, sections of all brain levels were analyzed with a Leitz optic microscope (Dialux 20 EB; Leica, Italy; 16X magnification; 25°C) and captured via Leica Imaging software in order to identify neuronal cell groups according to previous works [63]. Cell counting of telencephalon (TEL), hypothalamus (HTH), optic tectum (Te) and cerebellum (Cb) was performed on five sections/area by using the following formula: Ns = [Σ(N/Vsection)/n] x Vref, as previously reported for neuronal quantification in lungfish [4], where Ns = stained neurons per area; N = stained neurons/single section; Vsection = section volume; n = sections per area; Vref = volume of brain area.

### *Statistical analysis*

The quantification of apoptotic cells (TUNEL) in aestivating animals with respect to FW was performed using a one-way ANOVA followed by a *post hoc* Newman-Keul’s test when a *p*-value <0.05. Data deriving from qPCR analysis were evaluated by using a one-way ANOVA followed by Student’s *t* test. For *in situ* hybridization method, mRNA levels (OD ± s.e.m.) were expressed as % of control (FW) for the different brain regions and evaluated by using a one-way ANOVA followed by a *post hoc* Newman-Keul’s test.

## Ethical standards

Experimental protocols of this study were approved by the National University of Singapore Institutional Animal Care and Use Committee (IACUC Permits 813/05, S06/06 A and 035/09) and by MIUR (Italian University Research Ministry).

## Abbreviations

AMPA: α-amino-3-hydroxy-5-methyl-4-isoxazole-propionic acid; Cb: cerebellum; Cc: corpus cerebelli; Dp: dorsal pallium; FW: freshwater controls; HSPs: heat shock proteins; HIF-1α: hypoxia-inducible factor-1 alpha; Hyd: dorsal hypothalamus; HTH: hypothalamus; Hyv: ventral thalamus; In: intercalate nucleus; Lp: lateral pallium; OD: optical density; Pl: lateral portion of medial pallium; Pd: dorsal portion of medial pallium; Pv: ventral portion of medial pallium; Ri: pars intermedia of reticular nucleus; Sl: lateral subpallium; Sm: medial subpallium; Te: optic tectum; TEL: telencephalon; Thd: dorsal thalamus; Thv: ventral thalamus; 6dAE: 6 days of aerial normoxic aestivation; 6mAE: 6 months of aerial normoxic aestivation; 6dMUD: 6 days of mud hypoxic aestivation; 6mMUD: 6 months of mud hypoxic aestivation.

## Competing interests

The authors declare that they have no-financial competing interests in relation to this MS.

## Author’s contributions

GG design the study, carried out molecular studies, data analysis and drafted the MS; MZ participated in sequence alignment and data analysis; RMF participated in design of the study and statistical analysis; SFC and YKI participated in the design of study and in initial experiments on animal treatment and aestivating conditions; MC participated in design and coordination of the study and cooperated in the draft and final revision of the MS. All authors read and approved the final manuscript.
